# A Hybrid Water Balance Machine Learning Model to Estimate Inter-Annual Rainfall-Runoff

**DOI:** 10.3390/s22093241

**Published:** 2022-04-23

**Authors:** Amir Aieb, Antonio Liotta, Ismahen Kadri, Khodir Madani

**Affiliations:** 1 Laboratory of Biomathematics, Biophysics, Biochemistry, and Scientometric (BBBS), Bejaia University, Bejaia 06000, Algeria; amir18informatique@gmail.com (A.A.); madani28dz2002@yahoo.fr (K.M.); 2 Faculty of Computer Science, Free University of Bozen-Bolzano, 39100 Bolzano, Italy; 3 Department of Civil Engineering and Hydraulics, 8 May 1945 Guelma University, Guelma 24000, Algeria; kadri.ismahen@univ-guelma.dz; 4 Research Center of Agro-Food Technologies (CRTAA), Bejaia 06000, Algeria

**Keywords:** rainfall runoff, watershed, climate floor, modeling, water balance models, machine learning, multiple regression, decision tree

## Abstract

Watershed climatic diversity poses a hard problem when it comes to finding suitable models to estimate inter-annual rainfall runoff (IARR). In this work, a hybrid model (dubbed MR-CART) is proposed, based on a combination of MR (multiple regression) and CART (classification and regression tree) machine-learning methods, applied to an IARR predicted data series obtained from a set of non-parametric and empirical water balance models in five climatic floors of northern Algeria between 1960 and 2020. A comparative analysis showed that the Yang, Sharif, and Zhang’s models were reliable for estimating input data of the hybrid model in all climatic classes. In addition, Schreiber’s model was more efficient in very humid, humid, and semi-humid areas. A set of performance and distribution statistical tests were applied to the estimated IARR data series to show the reliability and dynamicity of each model in all study areas. The results showed that our hybrid model provided the best performance and data distribution, where the R^2^_Adj_ and *p*-values obtained in each case were between (0.793, 0.989), and (0.773, 0.939), respectively. The MR model showed good data distribution compared to the CART method, where *p*-values obtained by signtest and WSR test were (0.773, 0.705), and (0.326, 0.335), respectively.

## 1. Introduction

The irregular distribution of water resources in Mediterranean countries has been one of the most observed problems during the past twenty years, due to the great inter-annual variability of precipitation, seasonal rainfall regimes, summer drought, and intense precipitation [1]. In this area, several scientific studies have predicted a change in water balance states due to climate change, irregular water demand by different sectors, and poor management in the distribution to agricultural areas [2,3]. Underground water is a form of hydraulic resource, which crosses the soil surface. This depends mainly on precipitation and actual evapotranspiration [4]. Rainfall-runoff modeling helps us to determine the distribution of water accumulation on surfaces, which are characterized by geomorphological and climatic diversity, to understand the hydrological phenomena, and to visualize the state of the water system due to changes in permeable surfaces, vegetation, and climatic events. Rainfall-runoff estimation is a very complex area of study—it requires knowing the interconnection between several variables, which have a relationship with actual evapotranspiration, such as the climatic characteristics of the watershed, vegetation, water storage capacity, basin morphology, and meteorological parameters [5].

In the literature, water balance models used for inter-annual estimation are classified into three categories: empirical, physical, and conceptual [6]. The empirical models are non-linear and use artificial intelligence techniques, such as black boxes [7]. These models do not represent any relation to the physics of the watershed. On the other hand, they can effectively perform water estimation in ungauged watersheds. The physical models require a set of physical variables using a spatial-temporal scale to calibrate model parameters and to define a more dynamic model. An inconvenience is the difficulty of application due to data availability problems. On the other hand, conceptual models are the easiest type, which uses as input data climatic variables (e.g., rainfall, temperature, and potential evapotranspiration) without considering the spatial variability of watersheds. Most of these models are local and limited in their application when considering climatic conditions. The first models developed to estimate IAE_a_ were proposed by Schreiber [8] and Ol’Dekop [9], which involved a simple relationship between real evapotranspiration (E_a_), potential evapotranspiration (E_o_), and rainfall (R). Later, Budyko [10] proposed an average model to minimize the estimation errors obtained by Schreiber and Ol’dekop for different watershed responses. Certain models have been obtained by Boudyko curve derivation, which have shown a relationship between water and energy according to the following ratio: Ea/R and E_o_/R [11]. These models define another category called ‘conceptual parametric models’, as presented by Sharif [12] and Yang [13], in which the response equation has parameters obtained locally, depending on climatic characteristics and the storage capacity of the watershed.

The choice of an efficient and reliable model to assess inter-annual rainfall runoff (IARR) in regions characterized by great climatic variability is a more frequent problem in the literature. This study proposes a dynamic and flexible model for climatic characteristics of watersheds using machine learning techniques, applied to several climatic regions. The latter help to generalize the proposed model by uniform classification of input data into standard intervals. In the experimental component, we chose the northern Algeria region to define the hybrid model according to the climatic diversity which characterizes the area. We applied and compared a set of parametric and non-parametric conceptual models to 16 watersheds, classified into five bioclimatic floors: very humid, humid, semi-humid, Mediterranean, and semi-dry. The best models that demonstrated good performance on each climatic floor were used as input variables in the MR (multiple regression) and CART (classification and regression tree) machine learning. Finally, a new MR-CART hybrid model is presented in the form of a flowchart, which illustrates the necessary steps.

This article is organized into three basic sections. Following this introduction, we present the materials and methods used and provide details about the data and its context. Then, we introduce the machine learning models, present the experimental results, and draw conclusions.

## 2. Material and Methods

### 2.1. Study Area and Data

**Figure 1:**
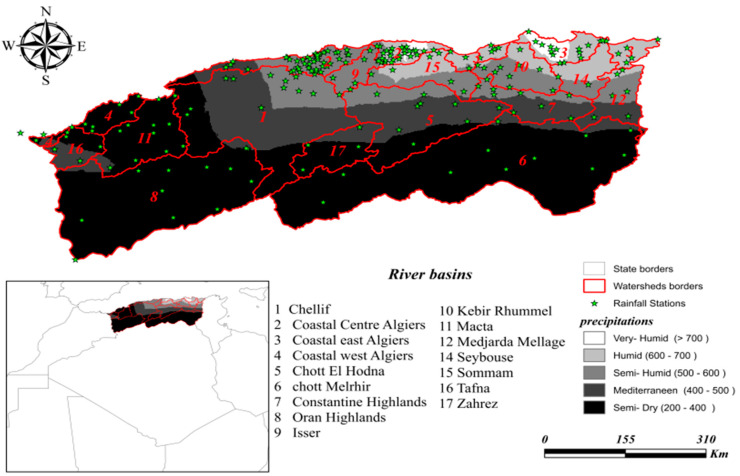
Map of northern Algeria area showing weather stations in different climate floors.

The northern Algeria region is one of the most important regions in the north of Africa, with an area of 480,000 km^2^, bordered on the north by the Mediterranean Sea, on the south by the northern Sahara, on the west by Morocco, and on the east by Tunisia. It is located between a longitude of −2.21 and 8.86 and a latitude of 32.75 and 37.1 [14]. Data used in this study was provided by 102 hydro-climatic stations, which are distributed over 17 watersheds, numbered from 1 to 17, excluding the basin numbered 13, which represents the Saharan region. As shown in Figure 1, the area has a climatic diversity classified into five climatic floors, from very humid to semi-dry. Between 1960 and 2020, the mean precipitation showed values which varied spatially between minimum values of 200 mm and maximum values of more than 700 mm. The very humid area covers the northern part of basins 3 and 2. On the other hand, the humid region is represented by the rest of basins 2 and 3, and the northern part of basins 12, 14, 10, and 15. The semi-dry climate floor is represented by basin 9, the middle area of basin 12, the southern part of basins 14, 10, 15, the western part of basin 2, and the northeast part of basin 1. The Mediterranean area is represented by the southern part of basins 12, 7, 16, the middle area of basins 5 and 1, and the northern part of basins 17. The southern region represents the semi-dry area, which covers basins 6, 8, 1, and 4, the southern part of basins 5, 17, 1, and the eastern area of basin 16.

The dataset used for this modeling was spatially obtained from 102 hydro-climatic stations between 1960 and 2020, using the inter-annual time scale which is the inter-annual rainfall (IAR), the inter-annual potential evapotranspiration (IAE_o_), and the real inter-annual rainfall-runoff (IARR). The independent variables of each sub-model used in this study were IAR and IAE_o_, which also represent the input data to the proposed model, obtained from the measurement history of the Algerian National Agency for Hydrological Resources (ANRH), the National Environmental Information Centers (NCEI-NOAA) https://www.ncei.noaa.gov/, accessed on 12 May 2021, and the climate knowledge portal https://climateknowledgeportal.worldbank.org/, accessed on 17 May 2021. Furthermore, the real IARR was used in this study as a response variable (dependent) in the machine learning and regression models, and to compare and verify the reliability of the proposed model in each bioclimatic area. The latter was obtained by reading the rainfall-runoff maps that are provided by the ANRH service.

### 2.2. Water Balance Model

In this section, a set of non-parametric and empirical models are used to propose a dynamic and reliable estimation of actual evapotranspiration (IAE_a_) on the inter-annual time scale. Estimating IAE_a_ helps to quantify the quantity of IARR according to the water balance, as represented by Equation (1) [15], which controls the amount of input and output water in a watershed, in the form of IAR, IAE_a_, IARR, and the change in water storage (ΔS), where ΔS is considered to be negligible.

(1) $$\mathrm{IAR}={\mathrm{IAE}}_{a}+\mathrm{IARR}+\Delta S\;$$

(2) $$\mathrm{IARR}=\mathrm{IAR}-{\mathrm{IAE}}_{a}$$

The different E_a_ models which were analyzed, and for which performance was compared on five bioclimatic floors in northern Algeria between 1960 and 2020, are as follows:

#### 2.2.1. Schreiber

Schreiber [8] proposed a simple exponential as represented by Equation (3), which shows the relationship between actual inter-annual evapotranspiration (IAE_a_) in terms of inter-annual precipitation (IAR) and mean annual potential evapotranspiration (IAE_o_).

(3) $${\mathrm{IAE}}_{a}=\mathrm{IAR}\times \left[1-\exp\left(-\frac{{\mathrm{IAE}}_{o}}{\mathrm{IAR}}\right)\right]$$

#### 2.2.2. Ol’Dekop

Ol’Dekop [9] used a trigonometric hyperbolic tangent function to show the relationship between the mean annual potential evapotranspiration (IAE_o_) and the drying factor (Q), which represents the ratio between IAE_o_ and IAR, where the equation of this model is as follows:

(4) $${\mathrm{IAE}}_{a}={\mathrm{IAE}}_{o}\times \left[\mathrm{Tan}h\;\left(Q\right)\right]$$

where, $Q=\frac{\mathrm{IAR}}{{\mathrm{IAE}}_{o}}$.

#### 2.2.3. Pike

This equation is a simple formula derived from the Turk model [16], where it is proposed that replacing the value 0.9 by 1 gives a better result [17]. The model formula is as follows:

(5) $${\mathrm{IAE}}_{a}=\frac{\mathrm{IAR}}{{\left[1+{\left(\frac{\mathrm{IAR}}{{\mathrm{IAE}}_{o}}\right)}^{2}\right]}^{0.5}}$$

#### 2.2.4. Budyko

Budyko [10] applied a geometric mean between Schreiber [8] and Ol’dekop [9], on the basis that the Schreiber model gives a result lower than the real data, while Ol’dekop’s estimation shows higher values, to give much better results (6).

(6) $${\mathrm{IAE}}_{a}={\left[\mathrm{IAR}\times \left[1-\exp\left(-\frac{{\mathrm{IAE}}_{o}}{\mathrm{IAR}}\right)\right]\times \mathrm{ETR}-\tan h\left(\frac{\mathrm{IAR}}{{\mathrm{IAE}}_{o}}\right)\right]}^{0.5}$$

#### 2.2.5. Yang

Yang [13] proposed an alternative model (7) to estimate the mean annual actual evapotranspiration using Budyko’s hypothesis, in which an adjustable parameter was introduced which can use the watershed characteristics and give a better estimation.

(7) $${\;\mathrm{IAE}}_{a}=\left[{\left[\left[{\left(\frac{{\mathrm{IAE}}_{o}}{\mathrm{IAR}}\right)}^{-A}\right]+1\right]}^{-\frac{1}{n}}\right]\times \mathrm{IAR},\;\mathrm{where}\;n\;>\;0$$

#### 2.2.6. Sharif

This is an improvement of the Mezentsev–Choudhury–Yang (MCY) model which replaces the b, k, and n parameters of the MCY equation with values 0, 2, and 1, respectively [12,18].

(8) $${\mathrm{IAE}}_{a}=\frac{2\times \mathrm{IAR}\times {\mathrm{IAE}}_{o}}{\mathrm{IAR}+2\times {\mathrm{IAE}}_{o}}$$

where IAR is the inter-annual rainfall, IAE_o_ is the inter-annual potential evapotranspiration, and IAE_a_ is the inter-annual actual evapotranspiration.

#### 2.2.7. Zhang

Zhang [15] proposed a relational model which uses simple interpolators between two water balance ratios (9) and (10), defined by Budyko [19]. These interpolators are also related to the mean potential evapotranspiration (IAE_o_) and plant available water content given by the coefficient (w). This relation is shown by Equation (11).

(9) $$\frac{R}{\mathrm{IAR}}\to 0,\;\frac{{\mathrm{IAE}}_{a}}{\mathrm{IAR}}\to 1,\;\frac{R_{n}}{\mathrm{IAR}}\to \infty$$

(10) $${\mathrm{IAE}}_{a}\to R_{n},\;\frac{R_{n}}{\mathrm{IAR}}\to A$$

(11) $$\frac{{\mathrm{IAE}}_{a}}{\mathrm{IAR}}=\frac{1+w\times \left(\frac{{\mathrm{IAE}}_{o}}{\mathrm{IAR}}\right)}{1+w\times \left(\frac{{\mathrm{IAE}}_{o}}{\mathrm{IAR}}\right)+{\left(\frac{{\mathrm{IAE}}_{o}}{\mathrm{IAR}}\right)}^{-1}}$$

where R is surface runoff, IAR is the mean annual rainfall, IAE_a_ is the mean actual evapotranspiration, and R_n_ is net radiation.

### 2.3. Machine Learning Models

#### 2.3.1. Multiple Regression Model (MR)

Regression is a graphical model, which expresses the goodness of fit between two or more sets of data. In hydro-climatic science, it is most frequently used for modeling, optimization, and comparative study between predictive and actual series. A simple regression illustrates the relationship between the dependent variable (Y) and the independent variable (X). In multiple regression, more than one independent variable (X_i_) can have a relationship with the dependent variable (Y) [20]. This relationship can be linear (MLR), or non-linear (MNLR) [21]. The least-squares method is used to estimate the model coefficients. The MLR equation is defined as follows:

(12) $$Y=\alpha +\sum_{i=1}^{n}\beta_{i}X_{i}+Ɛ$$

where α is the intercept, β_i_ is the regression coefficient, and Ɛ is the regression residual.

The subset problem is related to the choice of the selected variables or the best regression model. It involves using the set of n observations and m explanatory variables to build efficient multiple regression models by reducing the model trend errors. In the literature, the choice of a subset of explanatory variables is based on the objective function, which measures the efficiency of the model by balancing the number of explanatory variables used and the adjustment error according to several criteria, such as R^2^_Adj_, MSE, and Mallows’ Cp, etc. [22].

#### 2.3.2. Classification and Regression Tree Model (CART)

The CART model is a non-parametric procedure to predict continuous dependent variables with categorical and/or continuous predictor variables. This method is used in many fields [23,24,25]. In this model, the data is partitioned into nodes based on the conditional binary responses to questions that include the predictor variable y.CART models use a binary tree to divide the predictor space recursively into subsets in which the distribution of y is successively more homogeneous [26]. The decision tree is constructed using automatic stepwise variable selection to identify mutually exhaustive and exclusive subgroups of a population [27]. In the first step, the method selects the best optimum breakpoint in which the dependent variable may be separated into two groups. Then, each of the two resulting groups is further separated into two other subsets. Following this logic, the method generates a tree structure in which the dependent variable is optimally divided into a certain number of groups, which are characterized by maximum internal homogeneity and maximum external differentiation [28]. In modeling, CART uses a set of techniques for structuring data clusters, such as AID and CHAID [29]. The tree defines a set of rules for each node followed by its predicted value. In each estimation, the model verifies if the independent variable X_i_ accepts the clause, beginning from the child rules node to a father rules node to give the predicted value. A defect of this model is the possibility of having redundancy values.

## 3. Results

The progressive steps of modeling are described below in three core sections, starting with the statistical description of the elementary data series used in each sub-model, followed by comparison and performance analysis to choose the best water balance models used to generate the input variables, as well as the hybrid model design (dubbed MR-CART).

### 3.1. Data Description

The dataset used in this study contained 212 lines and 3 columns represented by IAR, IAE_o_, and IARR variables, which were observed by 212 watersheds. The dataset was processed to give a comparative view of the variability and data distribution between the input and response variables used in the modeling. This analysis is shown in Table 1 by a set of statistical parameters applied to unclassified and classified data using bio-climatic classes. According to the table, 45/212 data lines belonged to the semi-dry region, 16/212 data lines were obtained from the Mediterranean region, 15/212 data lines belonged to the semi-humid area, 11/212 data lines represented the humid region, and 15/212 data lines were obtained from the very humid class. The results showed great variability of IARR, which was observed in all the northern Algeria areas, given by a CV equal to 1.181. The actual IARR measurement showed a non-stationary distribution around the mean, where the median was less than the mean, which was given by values of 39 and 81.719, respectively. A total of 75% of these data lines had values less than the center of the value interval (AV), which equaled 252. This set of data varied between 7 and 104.3. In this area, the IAR input variable showed greater variability compared to IAE_o_, where the CV equaled 0.404 and 0.073, respectively. A total of 75% of IAR data lines ranged from 222 to 600, which belonged to the inter-annual aridity classes of semi-humid, Mediterranean, and semi-dry. On the other hand, the IAE_o_ provided stationary values, which were distributed around the mean, where the median and the mean were very close and equal to 1357.500 and 1351.598, respectively. A large change in variability and non-stationary distribution pose big problems in modeling reliability where data classification is required. To address this, we have represented the data series according to aridity classes. In the five data classes, the CV values showed no large variability.

For the IARR series, the CV provided values between 0.149 and 0.404, which were lower than 1.181 when we used the data series of northern Algeria. A decrease in variability was also observed by IAE_o_ and IAR in each climatic class, where the values of CV that were obtained for each data set were lower than 0.073 and 0.404, respectively. According to this classification, the data distribution of each variable used in the water balance models was stationary around the mean. Table 1 shows that the median and the mean for each variable were very close for each sub-series.

### 3.2. Experimental Results

**Figure 2:**
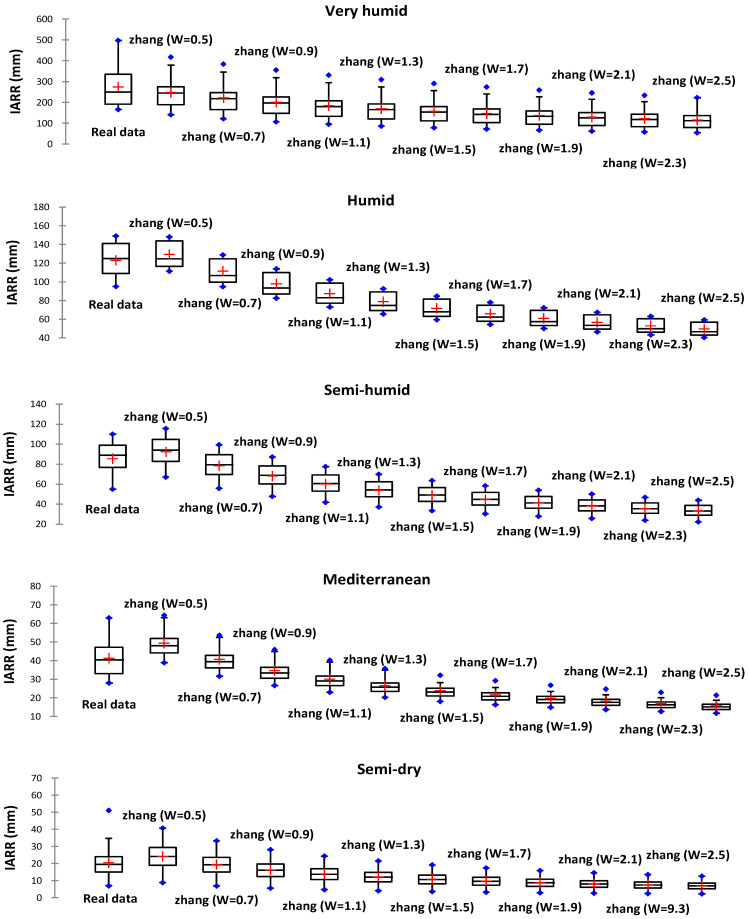
Boxplots of real and predicted IARR data series obtained by Zhang’s model in five climatic regions in northern Algeria for ‘w’ between 0.5 and 2.5.

**Figure 3:**
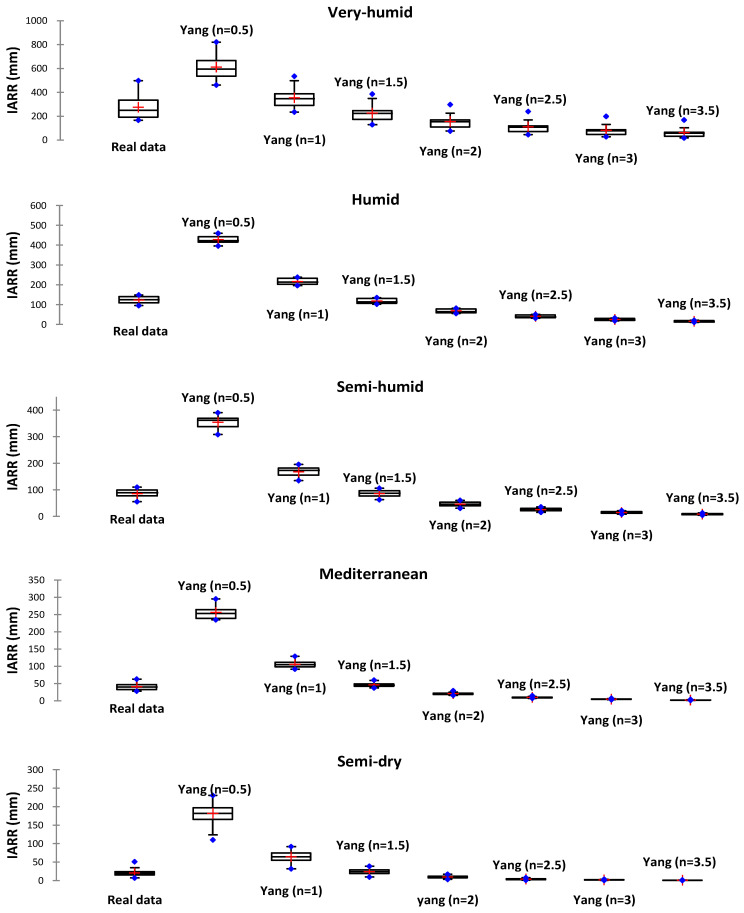
Boxplots of real and predicted IARR data series obtained by Yang’s model in five climatic regions in northern Algeria for ‘n’ between 0.5 and 3.5.

In this section, the set of water balance models presented above was compared and their performance in estimating IARR in five bioclimatic regions in northern Algeria between 1960 and 2020 was analyzed. We used boxplots and a set of performance tests including R^2^ [30], R^2^_Adj_ [31], MAE, and RMSE [32] to compare the distribution and variability of the predicted and actual IARR data for each model. Regression graphs and residual analysis were applied to determine the degree of fit between the data, and the modeling performance obtained by MR and CART machine learning. The hybrid model dynamicity is described in the next section, and the *t*-test and the *z*-test are introduced to analyze the significance of differences between the actual and predicted values of IARR in the whole study area using the different estimation models. This analysis showed the importance of the aridity factor in the estimation of underground water in large surface areas. As the first step in this study, we used the best W and N parameters proposed by the Zhang and Yang model in each bioclimatic area. According to the literature, the range values for the two parameters are as follows: W ∈ [0.5, 2.5], and N ∈ [0.5, 2.5]. Figure 2 and Figure 3 show the predicted data distribution of the Zhang and Yang model obtained values of W and N in boxplot form, which represent graphically the min, max, 1st Q, median, and 3rd Q values of each subset.

The results given in Figure 2 show that the best value of W of 0.5 was obtained in the very humid, humid, and semi-humid areas. In the Mediterranean and semi-dry region, the W parameter was 0.7; in each climatic area, the mean and median value of the predicted data series obtained for the best W was closer to that of the real series. Zhang’s model provided a more divergent estimation when W was more than 0.7, where the predicted values were lower than the actual data.

Table 2 shows the Zhang model performance using different W values in each climatic region. In the very humid and semi-humid areas, the R^2^ and the R^2^_Adj_ showed the greatest performance when W equaled 0.5; in addition, the MAE and the RMSE had minimum errors compared to the other cases. In the humid areas, the best W value was 0.5, as the R^2^_Adj_, MAE, and RMSE showed the best results, which were equal to 0.792, 8.981, and 10.757, respectively. In the Mediterranean and semi-dry floors, the R^2^ and the R^2^_Adj_, MAE, and RMSE showed the best results when W equaled 0.7. For the Yang model, the best estimate was obtained when the parameter n was chosen as 1.5 in all the five climate areas (Figure 3), where the mean and median values given by the predicted series were closer to that of the measured data series. In all comparative cases, Yang’s model gave values above the real data for n equals 0.5 and 1. In contrast, when the n parameter was greater than 1.5, the predicted values were lower than the real IARR. In the very humid areas, the real data series had greater variability compared to the predicted data, where the median was less than the mean in the real dataset, and the range between the 1st Q and the 3rd Q was greater than the quartile variation range of the predicted data.

Table 3 shows that the maximum errors given by Yang’s model, when compared with the results of each climatic region for n equals 1.5, were obtained in the very-humid region, where the MAE and RMSE were equal to 63.289 and 77.753, respectively. The R^2^ and R^2^_Adj_ parameters showed that Yang’s model gave good performances in all northern Algeria areas for n equals 1.5 when compared with other values of n. However, the best performance was obtained in the semi-humid area, where the R^2^_Adj_ equaled 0.934. In the Mediterranean region, the model performed less well, as demonstrated by an R^2^_Adj_ equaling 0.508.

**Figure 4:**
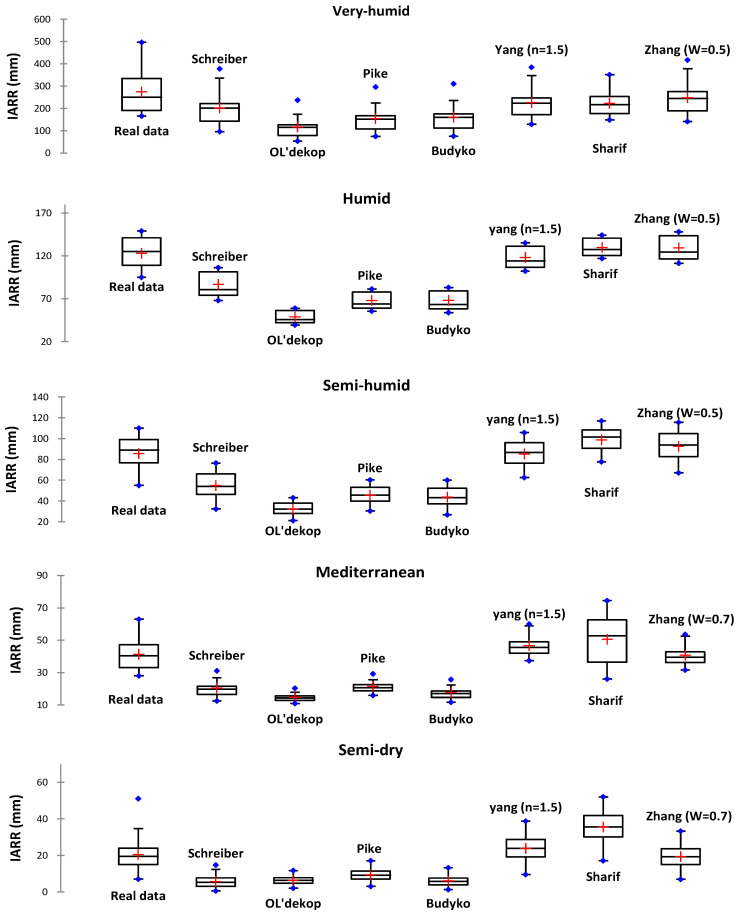
Box-plots of real and predicted IARR data series obtained by a set of non-parametric and empirical water balance models in five climatic regions of northern Algeria.

A pre-selection analysis of the best water balance models which were used to estimate the input data of the independent variable in the MR and CART machine learning model is presented in Figure 4 and Table 4. The figure shows a comparative graphical analysis of the predicted and actual data distribution using boxplots. In addition, Table 4 shows the results of the descriptive and performance tests of each model applied in each climatic area. The graphs show that in the very humid area, the IARR series obtained by the Schreiber, Yang, Sharif, and Zhang models gave a closer distribution to the real data when compared with the predicted data obtained by the Ol’dekop, Pike, and Budyko models. According to the graphical results, the data estimated by the Schreiber model was located below the actual data. The 1stQ, mean and median parameters showed that the best variability with real data was obtained by the Sharif, Yang, and Zhang models. Moreover, Table 4 shows that the best performance was given by the Sharif model, where the R^2^ and the R^2^_Adj_ equaled 0.775 and 0.757, respectively. The Schreiber, Yang, Sharif, and Zhang models gave a good estimate of IARR, where the MAE and the RMSE showed that the residual values were minimal compared to the other models. In the humid area, the four models showed the same behavior when using data observed in the very humid area. According to the graphs, the Schreiber and Zhang model provided the best data distribution with actual data.

On the other hand, the Yang model was more efficient than the Schreiber model. However, Table 4 shows that the best performance was obtained by the Zhang model, where the R^2^ and R^2^_Adj_ equaled 0.794 and 0.772, respectively. According to the error analysis, the Schreiber, Yang, Sharif, and Zhang models can be taken as candidate models to estimate input data used in MR and CART machine learning, where the MAE and the RMSE values are less than 25. However, the rest of the models showed a marked trend where the MAE and the RMSE values were above 55. The boxplots which represent the data obtained by the four models (Schreiber, Yang, Sharif, and Zhang) show a good distribution with the real data in the semi-humid areas. According to the 1st Q, mean, median, and 3rd Q parameters, the Zhang model gave the best estimate; as shown in Table 4 these parameters equaled 80.750, 92.477, 91.933, and 102.767, respectively.

The performance analysis showed that the best R^2^, R^2^_Adj_, MAE, and RMSE were also given by the Zhang model. However, the error analysis showed that the set of models can be accepted to estimate the input data in the IARR modeling where the MAE and RMSE do not exceed 15. On the other hand, with the Ol’dekop, Pike, and Budyko models the error MAE and RMSE was significant, being greater than 40. In the Mediterranean area, the Schreiber model had drawbacks in IARR estimation, where the R^2^ and R^2^_Adj_ equaled 0.407 and 0.364, respectively. The data predicted by this model had the same distribution compared to the Ol’dekop, Pike, and Budyko estimations. In the four models, the interval of variation of values was too small compared to the actual data, where the error given by MAE and RMSE was more than 20 (Table 4). According to the table, the four models gave a biased estimation, where the R^2^ showed values less than 0.45. In contrast, the graphs show that Sharif’s model gave a very high data distribution. The 1st Q, mean, median, and 3rd Q parameters demonstrated that the Zhang model gave the best distribution. Moreover, the performance analysis showed that the Yang, Sharif, and Zhang models performed well in estimating the IARR, where the R^2^ and R^2^_Adj_ values obtained by the three models were more than 0.60. The MAE and RMSE showed that these gave minimal errors compared to other models, where the residual values were less than 13 (Table 4). In semi-dry areas, the 1st Q, mean, median, and 3rd Q parameters showed that the best distribution of predicted data, when compared with the actual values, was obtained by the Yang and Zhang models (Figure 4 and Table 4). The Sharif model showed good variability and an estimate above the actual data series. According to Table 4, the R^2^ and the R^2^_Adj_ values showed that the best model was the Yang model, where the obtained errors were the minimum compared to the other models. The R^2^ showed that all the models can perform; however, the MAE, RMSE, and the statistical criteria of data variability showed that it is preferable to select the Yang, Sharif, and Zhang models to estimate IARR with machine learning.

#### Proposed Method

**Figure 5:**
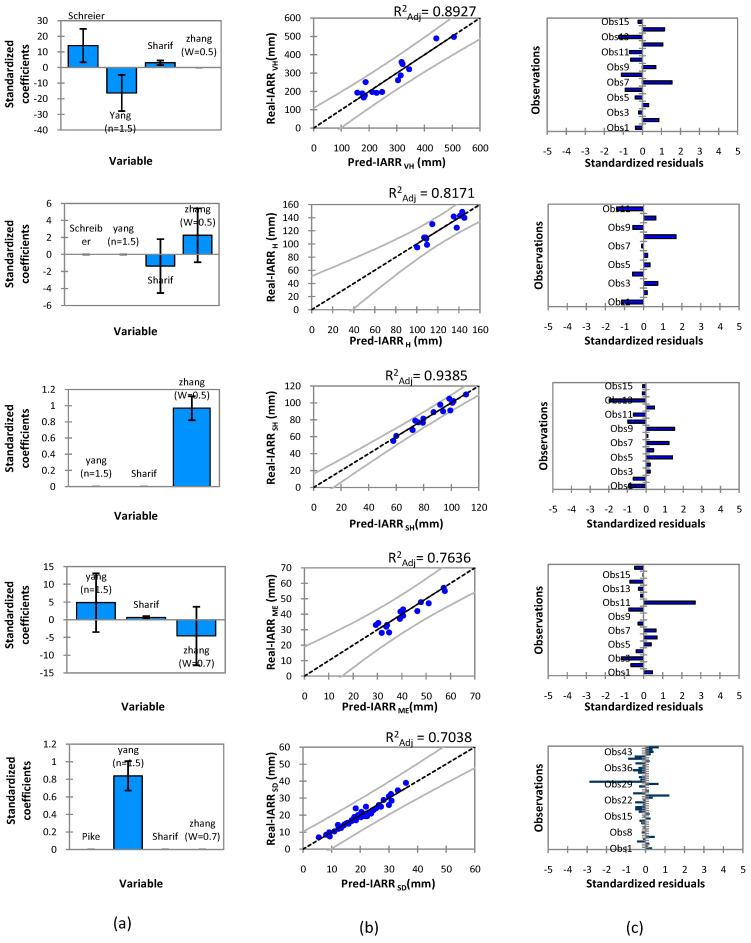
Graphs of (**a**) standardized coefficients, (**b**) regression, and (**c**) standardized residuals obtained by MR machine learning for IARR estimation in five climatic areas in northern Algeria. Predicted data (Pred), very humid (VH), humid (H). semi-humid (SH), Mediterranean (ME), semi-dry (SD).

The MR machine learning with the R^2^_Adj_ criterion was applied on subsets (X_i_) of the IARR predicted data which were obtained from the best non-parametric and empirical water balance models shown previously in Table 4 and Figure 4 on each climatic floor. The analysis steps and the graphical representation of this model were performed using the XLSTAT library, version 2018. The degree of fit between the predicted and the actual IARR data is shown in Figure 5 in the form of linear regression graphs. We have also graphically represented coefficients of each obtained trend model and the residuals standardized between the two series. According to the figure, the MR model showed the best performance compared to the water balance models selected previously. In the very humid area, the model showed a good adjustment of data where it belongs to the confidence range; moreover, the R^2^_Adj_ of the MR model proved its reliability compared to the Schreiber, Yang, Sharif, and Zhang models, which was 0.8927 (Table 4).

The model performed well when using the subsets obtained by the Schreiber, Yang, and Sharif models, where the standardized residuals were negligible being between −1.5 and 1.5 (Figure 5). The trend model obtained in this region is given as follows:

(13) $${\mathrm{IARR}\;\mathrm{VH}}_{\mathrm{MR}}=19.16{\times \mathrm{IARR}}_{\mathrm{Schreiber}}-24.31\times {\mathrm{IARR}}_{\mathrm{Yang}\left(n\;=\;1.5\right)}+5.63\times {\mathrm{IARR}\;}_{\mathrm{Sharif}\;}+639.52$$

In the humid areas, the MR model showed very good performance when using the input data (X_i_) obtained by the Sharif and Zhang (w = 0.5) models, where the R^2^_Adj_ equaled 0.8171 which showed the best performance compared to the selected water balance models given in Table 4. The model demonstrated the minimum errors, where the standardized residual values were [−2, 2]. In this case, the MR computational equation is as follows:

(14) $${\;\mathrm{IARR}\;H}_{\mathrm{MR}}=-2.40\times {\mathrm{IARR}}_{\mathrm{Sharif}}+2.97\times {\mathrm{IARR}}_{\mathrm{Zhang}\;\left(w\;=\;0.5\right)}+50.52$$

However, in the semi-humid region, the subset selection criteria showed that the MR model gave the best performance when using the data obtained by the Zhang model, where the estimation equation given by this machine learning is as follows:

(15) $${\;\mathrm{IARR}\;\mathrm{SH}}_{\mathrm{MR}}=1.09\times {\mathrm{IARR}}_{\mathrm{Zhang}\;\left(w\;=\;0.5\right)}-15.18$$

In this region, the predicted data showed good similarity with the measured values, as shown by the R^2^_Adj_ which equaled 0.9385. The use of the subsets which represented the predicted data obtained by the Yang, Sharif, and Zhang models in the MR model as an independent variable (X_i_) showed very good performance in the Mediterranean region, which was given by an R^2^_Adj_ equal to 0.7636. In the regression graph, the predicted and actual values showed a good fit; moreover, the standardized residual values indicated no trend (Figure 5). The model equation is given as follows:

(16) $${\;\mathrm{IARR}\;\mathrm{ME}\;}_{\mathrm{MR}}=7.92\times {\mathrm{IARR}}_{\mathrm{Yang}\;\left(n\;=\;1.5\right)}+0.43\times {\mathrm{IARR}}_{\mathrm{Sharif}}-7.77\times {\mathrm{IARR}}_{\mathrm{Zhang}\left(w\;=\;0.7\right)}-33.14$$

The predicted data series obtained by the Yang model in the semi-dry area proved to be the best subset that can be used in the MR model, in which the R^2^_Adj_ parameter gave the best value, equaling 0.7038. Moreover, the residual analysis showed a better error distribution where most of the values were between −1 and 1. The model equation is defined as follows:

(17) $${\;\mathrm{IARR}\;\mathrm{SD}}_{\mathrm{MR}}=1.04\times {\mathrm{IARR}}_{\mathrm{Yang}\;\left(n\;=\;1.5\right)}-4.41$$

**Figure 6:**
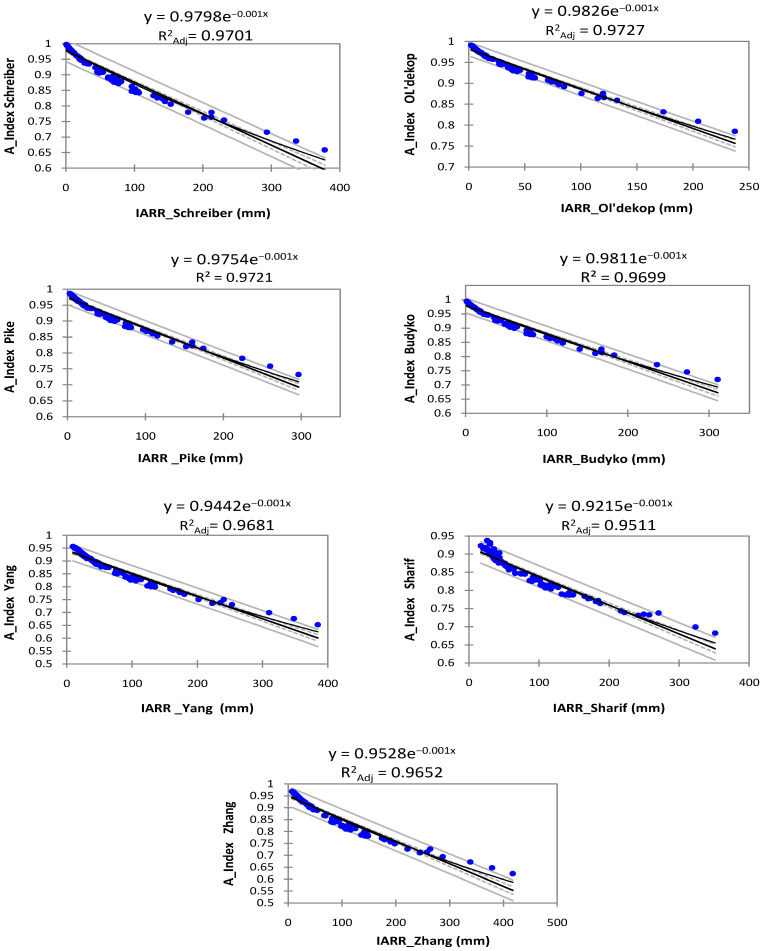
Graphs of non-linear regression between the A-Index data series and predicted IARR, which were obtained by the set of water balance models used in the northern Algeria region. Adjusted coefficient of determination (R^2^_Adj_).

Figure 6 shows a nonlinear relationship between the predicted IARR and the aridity index (A_Index) data obtained by each water balance model which was applied in all the northern Algeria areas. In this study, the A_Index series was obtained by Equation (18). In addition, the Prd-IARR variable was substituted by the A_Index in the next step using the trend equation given by each model in Figure 6 to change the subset bounds of each child node (Table 5 and Table 6).

This last characterized each climatic region and can make it easy to read the interval bounds for each node since the values are classified from min to max according to the most humid region to the driest, respectively.

(18) $$A\_\mathrm{Index}=\frac{{\mathrm{IAE}}_{a}}{\mathrm{IAR}}$$

In all cases, Figure 6 shows a good fit between the(A_Index) and the predicted IARR data series obtained by the non-parametric and empirical water balance models, where the R^2^_Adj_ showed very good values which varied between 0.9511 and 0.9727. Moreover, the regression graphs showed good similarity between the data in which all the values fell within the confidence ranges.

The conceptual steps of the decision tree and the predicted IARR data classification used in the CART model in each climate area are detailed in Table 5 and Table 6, where the set of parent and child nodes and the number of data (objects) used by each node is presented. The tables also present the set of estimation models which showed very good performance and a better classification of the independent variable (Prd-IARR) used in the CART non-parametric model. The conceptual results of the model in the very humid, humid, and semi-humid regions are given in Table 5. For the Mediterranean and semi-dry areas, the model structure is represented in Table 6. The Q parameter was obtained by multiplying A_Index values by 10^3^, which maintains the values classification and facilitates reading the bounds of each interval. It was also used in the formal algorithm of the model given in Table 7 to make it easier and more dynamic in application. Table 5 shows that in the very humid area, the CART model proposes a tree of two levels classified by the parent nodes numbered 1 and 4, obtained by the subset data given by the Zhang (w = 0.5) and Sharif models, respectively. In the humid zone, the Schreiber and Yang (n = 1.5) models showed very good performance, whereas the CART model showed a tree of two levels given by the parent nodes 1 and 2 in which 36.36% of the input data (X_i_) accepted the values obtained by the Schreiber model that was defined as follows: Q ∈ [828.610, 835.011]. However, the subset Q ∈ [835.011, 860.960] accepted the Q value 844.45 as an optimum boundary to subdivide this class into two subsets obtained by the Yang model. In the semi-humid area, the CART model accepted only the data given by the de Schreiber model as a subset (X_i_), where the proposed tree had only one level. Table 6 shows a tree of two levels given by the CART model in the Mediterranean area, where the Zhang (w = 0.7) and Sharif models showed the best data classification. Moreover, The Q subset of the Zhang model (Q ∈ [913.504, 923.161]) accepted another more efficient classification using Sharif’s data. In the semi-dry area, the CART model showed that the data obtained from the Sharif model provided a better classification, in which the tree structure of this model is given on one level and eight child nodes (Table 6).

The application steps of the CART model used to estimate the IARR in each climatic area are shown in Table 7 as a formal algorithm; the set of rules and estimated values (IARR-CART) corresponding to each child node are shown in the table. The algorithm execution needs only to read the tree from the child node to the parent node. For example, in the very humid region, to check if the Q value that was obtained by the Zhang model (Q_Zhang_) belongs to the interval [627.73, 723.36], it needs, as the first step, to check if there is another value Q obtained by the Sharif model (Q_Sharif_), in which the Q_Zhang_ and Q_Sharif_ can verify the clause defined by node 5 or 6. Where the two-child condition cannot be verified, the model uses the parent condition to ensure the belonging of the Q_Zhang_ value. In the end, the model gave the value 367.47 as the estimated result of IARR. The algorithm stops when the whole IARR series is estimated.

**Figure 7:**
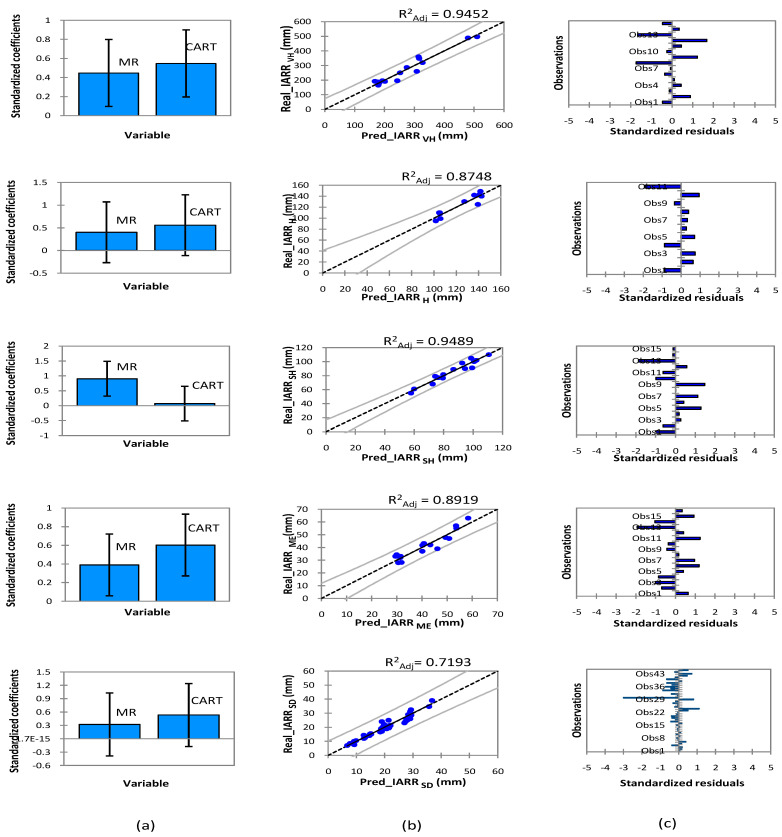
Graphs of (**a**) standardized coefficients, (**b**) regression, and (**c**) standardized residuals obtained by MR-CART’s hybrid model to estimate IARR in five climatic areas in northern. Predicted data (Pred), very humid (VH), humid (H), semi-humid (SH), Mediterranean (ME), semi-dry (SD).

The hybrid model’s performance, the degree of similarity between actual and predicted values, as well as the standardized residual analysis, are shown in Figure 7 for each climatic region. In the very wet area, the model showed excellent performance of data demonstrated by an R^2^_Adj_ equaling 0.9452 when the data subset estimated by the MR and CART model were used as independent variables in the multiple regression model used by the MR-CART model. In this area, the equation used to estimate IARR is defined as follows:

(19) $${\mathrm{IARR}\;\mathrm{VH}}_{\mathrm{MR}-\mathrm{CART}}=0.474\times {\mathrm{IARR}\;\mathrm{VH}}_{\mathrm{MR}}+0.5738\times {\mathrm{IARR}\;\mathrm{VH}}_{\mathrm{CART}}-13.1289$$

The hybrid model showed good performance in the humid region, where the R^2^_Adj_ equaled 0.8748. In the regression graph, no trend was observed by the model; moreover, the standardized residuals values were negligible, being between −2 and 1. The trend equation obtained by this model that is used to estimate IARR is as follows:

(20) $${\mathrm{IARR}\;H}_{\mathrm{MR}-\mathrm{CART}}=0.4426\times {\mathrm{IARR}\;H}_{\mathrm{MR}}+0.60636\times {\mathrm{IARR}\;H}_{\mathrm{CART}}-6.1320$$

In the semi-humid area, the subset selection criteria in the multiple regression model which was used to define the trend equation of the MR-CART model gave great importance to the dataset obtained by MR. The model showed a small improvement and good performance compared to previously applied machine learning in which all data showed a good fit in the regression graph, with all values falling within the confidence intervals. The MR-CART model equation is given as follows:

(21) $${\mathrm{IARR}\;\mathrm{SH}}_{\mathrm{MR}-\mathrm{CART}}=0.9322\times {\mathrm{IARR}\;\mathrm{SH}}_{\mathrm{MR}}+0.0717\times {\mathrm{IARR}\;\mathrm{SH}}_{\mathrm{CART}}-0.42176$$

In the Mediterranean area, the hybrid model showed very good performance compared to all the models previously applied, where the R^2^_Adj_ equaled 0.8919, showing more than 10% performance improvement. In this area, the mathematical equation of the model is defined as follows:

(22) $${\mathrm{IARR}\;\mathrm{ME}}_{\mathrm{MR}-\mathrm{CART}}=0.4467\times {\mathrm{IARR}\;\mathrm{ME}}_{\mathrm{MR}}+0.6578\times {\mathrm{IARR}\;\mathrm{ME}}_{\;\mathrm{CART}}-4.2848$$

A small improvement in the estimated IARR was observed by the hybrid model compared to the MR model in the semi-dry climatic floor, where the R^2^_Adj_ equaled 0.7193. The regression curve showed that no trend was given by this model. The equation is given as a function of the IARR_MR_ and IARR_CART_ variables, as follows:

(23) $${\mathrm{IARR}\;\mathrm{SD}}_{\mathrm{MR}-\mathrm{CART}}=0.3836\;{\times \;\mathrm{IARR}\;\mathrm{SD}}_{\mathrm{MR}}+0.6306\;{\times \;\mathrm{IARR}\;\mathrm{SD}}_{\;\mathrm{CART}}-0.2650$$

The comparative performance analysis of the three proposed models, which are MR, CART, and MR-CART is shown in Table 8 for each climatic region, where a set of statistical parameters was used to study the predicted IARR data distribution relative to the actual data. The results showed strong performance and good dynamicity of the hybrid model compared to the MR and CART model. In the very humid region, the R^2^ and R^2^_Adj_ showed that the greatest values were given by the MR-CART model, which equaled 0.9574 and 0.9452, respectively. On the other hand, the CART model performed better than the MR model. The variability analysis showed that the data series obtained by the hybrid model had a very similar distribution to the real data, where the SD given by the two series equaled 102.307 and 105.244, respectively. In the humid area, the hybrid model showed an improvement compared to the MR and CART models, where the R^2^ and R^2^_Adj_ equaled 0.886 and 0.875, respectively. In addition, the error for MAE and RMSE showed minimum values when compared to errors given by the other models. Moreover, the predicted data series obtained by the hybrid model showed a close variability to the real data, shown by an SD equal to 17.628.

The MR model was more efficient than the CART model showing a good distribution of data compared to the real values in the semi-humid area, which was given by an R^2^_Adj_ and an SD equal to 0.9385 and 15.2901, respectively. On the other hand, the hybrid model had the best performance, as shown by an R^2^_Adj_ equal to 0.949. The comparative study showed that the series obtained by this model had better variability compared with the real series. In addition, the RMSE and MAE errors showed that the MR-CART model gave minimal errors compared to the MR and CART models, respectively. The hybrid model also showed the best performance in the Mediterranean and semi-dry areas, as shown by R^2^_Adj_ equaling 0.892 and 0.719, respectively. However, in the semi-dry region, the series obtained by CART showed a high level of similarity with the predicted data of the hybrid model, where the SD showed a close variability obtained from the two series equaling 7.153 and 7.214, respectively. In addition, the residual analysis given by RMSE showed values equaling 4.570 and 4.521, respectively.

**Figure 8:**
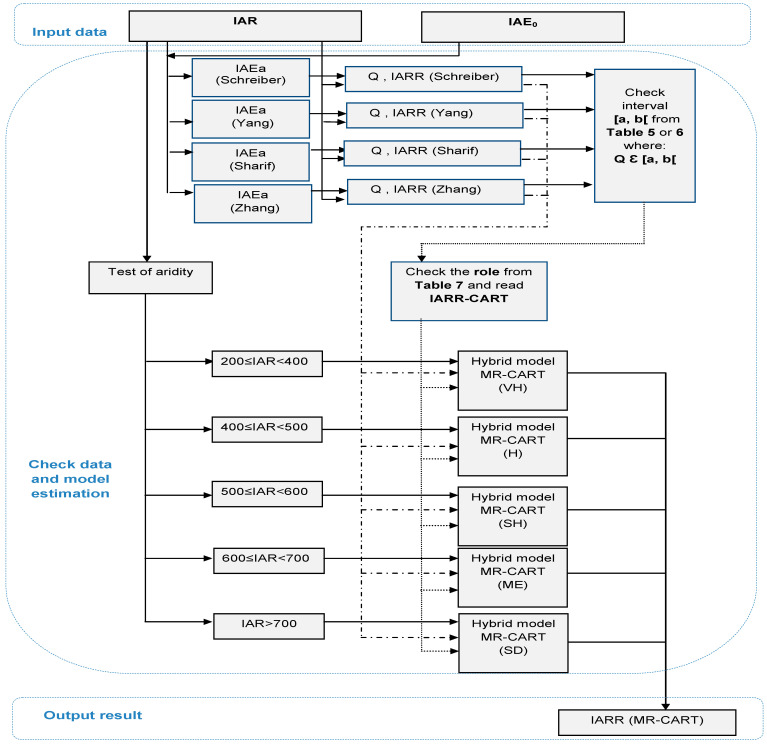
Flowchart summarizing steps design of MR-CART proposed model of IARR. Very humid (VH), humid (H), semi-humid (SH), Mediterranean (ME), semi-dry (SD), inter-annual rainfall (IAR), inter-annual potential evapotranspiration (IAE_o_), inter-annual actual evapotranspiration (IAE_a_).

The application steps of the MR-CART model are shown in Figure 8 in the form of a flowchart that expresses the operating dynamism, beginning with the input data selection and estimation through to obtaining the final results. The model is divided into three basic sections, which are given in the figure by input data, check data and model estimation, and output result. According to the figure, the model uses the IAR and the IAE_o_ as independent variables (X_i_) in the Schreiber, Yang, Sharif, and Zhang models to estimate IAE_a_ and Q. In the preprocessing step, the MR-CART model prepares the IARR_predicted_ and Q subsets for the next step. At each treatment, the model checks the climatic characteristics of the measuring station using the spatial classification of the IAR interval to select the corresponding equation of the MR-CART model. The model searches for the best rule given by the CART model which can verify the suitability of the Q value to generate the IARR predicted value (IARR_CART_). This last is used in the MR-CART equation. The process is recursive depending on the spatial sample size. Finally, the predicted dataset (IARR MR-CART) is given in the last section of the model as the final result.

## 4. Discussion

**Figure 9:**
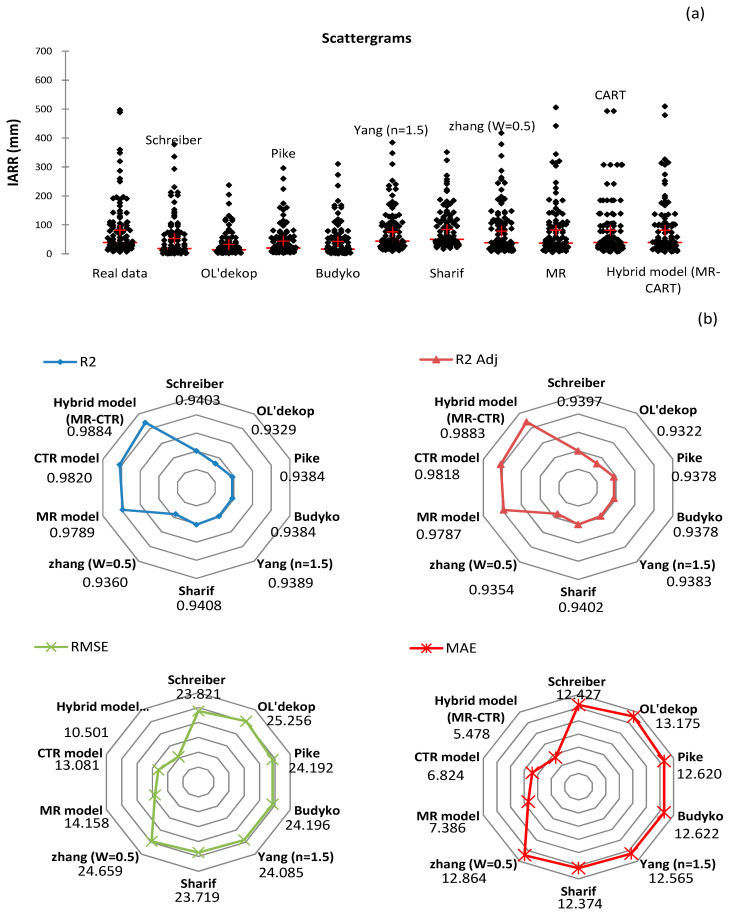
Graphs of (**a**) scattergrams and (**b**) radars showing data distribution and performance of proposed models in Algeria’s northern area. Coefficient of determination (R^2^), adjusted coefficient of determination (R^2^_Adj_), mean absolute error (MAE), root mean square error (RMSE).

A performance comparison of the proposed models with the non-parametric and empirical water balance models used in this study is shown in Figure 9 and Table 9. The models were applied in the northern Algeria area without taking into account the data classification of each climatic level. This allowed us to compare the residual trend and the dynamicity of each model in the large areas. The performance tests and the spatial distribution of the predicted and actual data are shown in the form of radars and scattergram graphs (Figure 9). In addition, a set of parametric and non-parametric tests, which were the *T*-test [33], Z-test [34], F-test [35], sign test [36], and WSRtest [37] were applied to verify if there were significant differences in the means, variance, and distribution between the real and predicted data for each model. The results showed that the best performance and distribution of predicted data compared to the actual values was obtained by the MR-CART hybrid model, with R^2^, R^2^_Adj_ shown in the graphs equaling 0.9884 and 0.9883, respectively. Moreover, the RMSE and MAE errors obtained by the model showed the smallest values, equaling 10.501 and 5.478, respectively. According to the performance tests, the CART model was placed in the second position compared to the other models (Figure 9). However, the scattergrams showed the model had drawbacks when compared to the real data distribution, as most of the predicted values obtained by the CART model were repetitive. Thus, it is more efficient to use the MR model. The latter showed good performance, as shown by R^2^ and R^2^_Adj_ equaling 0.9789 and 0.9787, respectively. In this study, all the non-parametric and empirical water balance models gave lower performance than the proposed models, where the R^2^ and R^2^_Adj_ were lower than 0.95. In addition, the RMSE and MAE showed that these models gave significant errors. Table 9 shows significant residuals were obtained by the Schreiber, Ol’dekop, Pike, and Budyko models, in which the parametric (e.g., *t*-test, *z*-test, *f*-test) and the non-parametric (e.g., sign test, WSR test) tests showed poor variability and data distribution; respectively, compared to actual data, where the *p*-values given by these tests were less than 0.05. On the other hand, Zhang’s model gave good data estimation compared to the Yang and Sharif models, where the *p*-value obtained by each test was between 0.209 and 0.447. However, the predicted data series given by both models showed no significant difference in variability with the actual data series, where the *t*-test, *z*-test, and *f*-test results obtained for the two models showed *p*-values of more than 0.05.

In comparison, the data distribution of the two series was poor, with the sign test and the WSR test showing *p*-values less than 0.05. According to Table 9, the proposed models (MR, CART, and MR-CART) were the most efficient and no significant difference was observed compared to the real IARR series, the *p*-values given by all tests being more than 0.5. The best model remained MR-CART, in which all the tests showed the best results and the data series obtained had very good similarity with the real dataset. In addition, the *p*-values obtained for the sign test and the WSR test showed that it is preferable to use the MR model as a second choice. The latter had better data distribution compared to the CART model despite its performance shown in Figure 9.

## 5. Conclusions and Future Work

The rainfall-runoff estimation, using an inter-annual time scale in a large area which is characterized by great climatic diversity, suffers from the problem of finding a better dynamic model adaptable to the spatial variability and the climatic conditions of the region. There are several models, but most are classified as non-parametric and empirical for local application, or are conceptual and physical and are difficult to apply due to dataset availability problems (such as vegetation index and watershed storage capacity). In this work, MR and CART machine learning was used to propose a dynamic model based on IARR predicted data as input data, obtained by a set of the most efficient water balance models in each climatic class in which both models applied the selection criteria to the input data subsets to give the best estimation. The experimental part of the modeling was applied in the northern Algeria area which is characterized by very humid, humid, semi-humid, Mediterranean, and semi-dry climates. A comparative study between water balance models in each climate floor showed that the Yang, Sharif, and Zhang models performed better throughout the northern Algeria area. It was shown that the choice of Yang’s parameter (n) equaled 1.5 giving the best performance in all the study areas. However, Zhang’s model showed excellent performance in the very humid, humid, and semi-humid areas when w equaled 0.5. Furthermore, the model gave good reliability in the Mediterranean and semi-dry areas when w equaled 0.7. In addition, the Schreiber model showed good performance in the very humid, humid, and semi-humid regions, where the R^2^_Adj_ varied between 0.667 and 0.928. In the five climatic classes, the performance analysis showed that the MR and CART model was more reliable compared to the water balance models used above, where, in the very humid region, the R^2^_Adj_ showed good performance for both models, shown by values of 0.8927 and 0.9101, respectively. This performance was also obtained in the humid region, where the R^2^_Adj_ equaled 0.8171 and 0.8450, respectively. In the semi-humid floor, the MR and CART model showed a small improvement compared to the previous models, where the input data subsets used in the two models were obtained by Zhang and Schreiber, respectively. In the Mediterranean and semi-dry areas, both machines showed a better performance as given by an R^2^_Adj_ equal to (0.7636, 0.7038) and (0.8382, 0.7137), respectively. The aridity data series (A_Index) showed good similarity with predicted data which was obtained by all the water balance models cited above, where the R^2^_Adj_ had values more than 0.95. This dataset was used by the CART model to generalize the data classification of each child node in the formal algorithm of the model. The MR model showed a better distribution of data compared to that obtained for the CART model, where the *p*-values for the sign test and the WStest equaled (0.773, 0.705) and (0.326, 0.335), respectively. According to the performance tests, the MR-CART hybrid model showed the best performance, where the R^2^_Adj_ had values between 0.793 and 0.989 in the five climatic classes, and 0.9883 in the northern Algeria region. In addition, the parametric and non-parametric tests (i.e., *t*-test, *z*-test, *f*-test, sign test, and WSRtest) showed that the hybrid model was dynamic and gave better variability and data distribution compared to the real data series, in which the *p*-values obtained by all the tests were between 0.7193 and 0.989.

Future work will seek to develop a forecasting model to estimate inter-annual rainfall runoff (IARR) using continuous and discontinuous hydro-climatic datasets. We would also like to observe the effect of the climatic indices on the spatial estimation of IARR.

## Figures and Tables

**Table 1:** Statistical description of the inter-annual dataset between 1960 and 2020 used in water balance modeling in five bioclimatic areas of northern Algeria.

Statistic	All Data			Very Humid			Humid			Semi-Humid			Mediterranean			Semi-Dry		
	IARR	IAE_o_	IAR	IARR	IAE_o_	IAR	IARR	IAE_o_	IAR	IARR	IAE_o_	IAR	IARR	IAE_o_	IAR	IARR	IAE_o_	IAR
N. data	102.000	102.000	102.000	15.000	15.000	15.000	11.000	11.000	11.000	15.000	15.000	15.000	16.000	16.000	16.000	45.000	45.000	45.000
Min	7.000	1180.000	222.000	166.000	1190.000	700.000	95.000	1185.000	610.000	55.000	1180.000	501.000	28.000	1195.000	400.000	7.000	1210.000	222.000
Max	497.000	1610.000	1107.000	497.000	1455.000	1107.000	149.000	1445.000	695.000	110.000	1460.000	598.000	62.950	1445.000	483.000	51.000	1610.000	394.000
Sum	8335.362	137,863.000	50,455.600	4118.860	19,551.000	13,117.000	1351.291	14,573.000	7187.000	1283.000	19,605.000	8367.000	661.055	21,127.000	6888.000	921.156	63,007.000	14,896.600
1st Q	20.625	1285.000	332.750	191.500	1237.500	783.500	109.000	1266.500	636.500	76.750	1222.000	535.500	33.022	1271.250	410.000	15.000	1350.000	309.000
Median	39.000	1357.500	415.500	250.000	1300.000	845.000	125.000	1340.000	650.000	89.000	1297.000	565.000	40.343	1345.000	425.500	19.500	1400.000	330.000
3rd Q	104.250	1410.000	607.000	334.500	1353.500	951.500	141.000	1407.500	669.500	99.000	1392.000	582.500	47.197	1366.750	442.750	24.000	1450.000	351.000
AV *	252.000	1395.000	664.500	331.500	1322.500	903.500	122.000	1315.000	652.500	82.500	1320.000	549.500	45.475	1320.000	441.500	29.000	1410.000	308.000
Mean	81.719	1351.598	494.663	274.591	1303.400	874.467	122.845	1324.818	653.364	85.533	1307.000	557.800	41.316	1320.438	430.500	20.470	1400.156	331.036
SD	96.510	98.801	200.100	105.200	72.300	119.700	18.400	85.400	24.200	15.800	91.700	31.900	10.100	76.800	26.700	8.300	96.900	37.400
CV **	1.181	0.073	0.404	0.383	0.055	0.137	0.1500	0.064	0.037	0.185	0.070	0.057	0.245	0.058	0.062	0.405	0.069	0.113

Inter-annual rainfall runoff (IARR), inter-annual potential evapotranspiration (IAE_o_), inter-annual rainfall (IAR), number of data (N. data), 1st quartile (1st Q), 3rd quartile (3rd Q), average (AV), std. deviation (SD),coefficient of variation (CV). * CV = SD/Mean. ** AV = (Min + Max)/2.

**Table 2:** Performance analysis of Zhang’s model in five climatic areas of northern Algeria, where w is between 0.5 and 2.5.

Climate Floor	Statistic	Zhang W = 0.5	Zhang W = 0.7	Zhang W = 1.7	Zhang W = 1.9	Zhang W = 2.1	Zhang W = 2.3	Zhang W = 2.5
Very humid	R^2^	0.685	0.685	0.677	0.675	0.673	0.671	0.668
	R^2^_Adj_	0.661	0.66	0.653	0.65	0.648	0.646	0.643
	MAE	45.688	67.002	146.652	137.439	145.245	152.412	158.804
	RMSE	65.489	80.273	157.022	147.515	161.08	168.074	174.361
Humid	R^2^	0.803	0.801	0.808	0.809	0.809	0.81	0.81
	R^2^_Adj_	0.792	0.777	0.787	0.787	0.788	0.788	0.789
	MAE	8.981	11.626	65.846	60.875	66.243	69.955	73.211
	RMSE	10.757	14.576	66.442	61.442	67.286	70.997	74.255
Semi-humid	R^2^	0.969	0.969	0.968	0.968	0.968	0.967	0.967
	R^2^_Adj_	0.967	0.966	0.965	0.965	0.965	0.965	0.965
	MAE	7.017	7.172	44.687	41.146	47.407	50.014	52.287
	RMSE	8.066	8.51	45.413	41.831	48.277	50.91	53.208
Mediterranean	R^2^	0.48	0.525	0.446	0.445	0.445	0.444	0.444
	R^2^_Adj_	0.441	0.516	0.407	0.406	0.405	0.404	0.404
	MAE	9.985	5.74	21.662	19.809	23.067	24.4	25.551
	RMSE	10.956	7.519	21.938	20.066	24.549	25.836	26.952
Semi-arid	R^2^	0.703	0.703	0.702	0.702	0.702	0.702	0.702
	R^2^_Adj_	0.696	0.696	0.695	0.695	0.695	0.695	0.695
	MAE	4.706	2.202	9.733	8.853	12.352	12.974	13.507
	RMSE	5.875	4.764	10.27	9.346	13.83	14.449	14.982

Coefficient of determination (R^2^), adjusted coefficient of determination (R^2^_Adj_), mean absolute error (MAE), root mean square error (RMSE).

**Table 3:** Performance analysis of Yang’s model in five climatic areas of northern Algeria, where ‘n’ was between 0.5 and 3.5.

Climate Floor	Statistic	Yang n = 0.5	Yang n = 1	Yang n = 1.5	Yang n = 2	Yang n = 2.5	Yang n = 3	Yang n = 3.5
Very humid	R^2^	0.669	0.684	0.690	0.692	0.691	0.689	0.685
	R^2^_Adj_	0.643	0.659	0.666	0.668	0.668	0.665	0.661
	MAE	336.917	79.873	63.289	123.699	164.240	193.151	212.914
	RMSE	342.734	99.480	77.753	136.170	177.727	205.967	225.710
Humid	R^2^	0.509	0.731	0.792	0.810	0.815	0.815	0.814
	R^2^_Adj_	0.455	0.701	0.769	0.789	0.794	0.795	0.794
	MAE	305.252	93.448	8.684	54.747	82.216	98.011	107.386
	RMSE	305.594	93.932	10.331	55.752	83.115	98.962	108.415
Semi-humid	R^2^	0.794	0.917	0.938	0.935	0.927	0.918	0.908
	R^2^_Adj_	0.778	0.910	0.934	0.930	0.922	0.912	0.901
	MAE	268.481	81.956	4.148	39.989	60.321	71.227	77.258
	RMSE	268.806	82.122	4.831	40.731	61.185	72.240	78.394
Mediterranean	R^2^	0.466	0.497	0.513	0.442	0.425	0.413	0.404
	R^2^_Adj_	0.428	0.462	0.508	0.402	0.384	0.371	0.362
	MAE	214.877	64.780	7.725	19.890	31.122	36.339	38.835
	RMSE	215.295	65.264	9.080	21.497	32.363	37.527	40.020
Semi-dry	R^2^	0.673	0.700	0.704	0.701	0.696	0.689	0.680
	R^2^_Adj_	0.665	0.693	0.697	0.694	0.689	0.681	0.673
	MAE	161.191	43.641	4.446	11.120	16.653	18.861	19.773
	RMSE	162.451	44.379	5.674	12.587	18.100	20.383	21.348

Coefficient of determination (R^2^), adjusted coefficient of determination (R^2^_Adj_), mean absolute error (MAE), root mean square error (RMSE).

**Table 4:** Statistical tests of data distribution and performance analysis of a set of non-parametric and empirical water balance models applied in five climate areas of northern Algeria.

Climate Floor	Statistic	Real Data	Schreiber	Ol’dekop	Pike	Budyko	Yang	Sharif	Zhang
Very humid	1st Q	191.500	142.853	79.234	108.015	111.792	172.325	177.104	189.116
	Median	250.000	201.822	114.986	152.211	159.778	223.041	216.700	244.418
	3rd Q	334.500	221.681	126.426	167.167	175.508	246.757	253.496	275.501
	Mean	274.591	202.740	117.382	154.542	161.413	226.406	222.811	248.345
	R^2^	1.000	0.690	0.672	0.662	0.690	0.690	0.775	0.685
	R^2^_Adj_	1.000	0.667	0.662	0.660	0.666	0.666	0.757	0.661
	MAE	0.000	84.731	157.209	123.699	118.372	63.289	61.615	75.688
	RMSE	0.000	93.259	171.808	136.170	129.444	77.753	80.017	85.489
Humid	1st Q	109.000	84.061	42.118	59.222	58.285	106.793	120.299	116.514
	Median	125.000	90.628	45.682	64.058	63.409	114.036	127.279	124.550
	3rd Q	141.000	101.443	56.376	77.984	79.338	131.099	140.595	143.678
	Mean	122.845	96.914	48.879	68.097	68.215	118.159	129.668	129.222
	R^2^	1.000	0.714	0.712	0.710	0.713	0.792	0.756	0.794
	R^2^_Adj_	1.000	0.693	0.691	0.689	0.693	0.769	0.729	0.772
	MAE	0.000	24.930	73.966	54.747	54.629	8.684	10.912	8.081
	RMSE	0.000	24.863	74.958	55.752	55.488	10.331	12.595	10.057
Semi-humid	1st Q	76.750	46.232	27.957	39.717	37.178	76.460	90.785	80.750
	Median	89.000	73.893	32.040	45.516	43.077	86.578	101.400	91.933
	3rd Q	99.000	86.040	37.764	53.127	52.107	96.049	108.303	102.767
	Mean	85.533	74.706	32.188	45.544	43.579	85.132	98.614	92.477
	R^2^	1.000	0.928	0.934	0.935	0.930	0.935	0.927	0.939
	R^2^_Adj_	1.000	0.922	0.929	0.930	0.925	0.930	0.921	0.933
	MAE	0.000	14.828	53.345	40.989	41.954	4.148	13.081	3.017
	RMSE	0.000	14.199	54.232	40.731	42.519	5.831	14.285	4.066
Mediterranean	1st Q	33.022	16.436	12.819	18.638	14.631	41.936	36.491	36.150
	Median	40.343	19.794	14.250	20.648	16.972	45.478	52.701	39.429
	3rd Q	47.197	21.590	15.535	22.503	18.518	48.959	62.517	42.789
	Mean	41.316	20.432	14.798	21.426	17.626	46.576	50.516	40.699
	R^2^	1.000	0.407	0.440	0.442	0.418	0.616	0.697	0.612
	R^2^_Adj_	1.000	0.364	0.400	0.402	0.377	0.608	0.675	0.603
	MAE	0.000	20.884	26.518	19.890	23.690	7.725	11.118	5.740
	RMSE	0.000	22.330	27.882	21.497	25.060	9.080	12.769	7.519
Semi-dry	1st Q	15.000	3.064	4.741	6.999	3.904	19.140	25.135	15.032
	Median	19.500	5.274	6.282	9.241	5.778	23.808	28.588	19.284
	3rd Q	24.000	7.708	7.839	11.504	7.638	28.714	36.772	23.622
	Mean	20.470	5.641	6.366	9.350	6.004	23.856	28.543	19.376
	R^2^	1.000	0.679	0.701	0.701	0.690	0.706	0.701	0.703
	R^2^_Adj_	1.000	0.671	0.694	0.694	0.683	0.700	0.694	0.696
	MAE	0.000	14.830	14.104	13.120	14.466	4.446	11.481	5.202
	RMSE	0.000	15.959	15.566	14.587	15.748	5.674	12.775	6.764

1st quartile (1st Q), 3rd quartile (3rd Q), coefficient of determination (R^2^), adjusted coefficient of determination (R^2^_Adj_), mean absolute error (MAE), root mean square error (RMSE).

**Table 5:** Structure of CART decision tree which is applied for modeling IARR in the very humid, humid, and semi-humid areas in northern Algeria.

Climate Floor	*p*-Value	Objects	%	Parent Node	Sons Node	W.B.M *	IARR (W.B.M)	A-Index **	Q ***
Very humid	0	15	100.00%						
	0	6	40.00%	1	2	Zhang (W = 0.5)	[141.207, 209.585]	[0.772, 0.827]	[772.652, 827.330]
	0	5	33.33%	1	3	Zhang (W = 0.5)	[209.585, 275.501]	[0.723, 0.772]	[723.360, 772.652]
	0.031	4	26.67%	1	4	Zhang (W = 0.5)	[275.501, 417.300]	[0.627, 0.723]	[627.731, 723.360]
	0	2	13.33%	4	5	Sharif	[249.605, 296.940]	[0.684, 0.717]	[684.763, 717.950]
	0	2	13.33%	4	6	Sharif	[296.940, 351.434]	[0.648, 0.684]	[648.441, 684.763]
	0.0225	11	100.00%						
	0.0033	7	63.64%	1	2	Schreiber	[67.956, 98.557]	[0.835, 0.860]	[835.011, 860.960]
Humid	0	4	36.36%	1	3	Schreiber	[98.557, 106.253]	[0.828, 0.835]	[828.610, 835.011]
	0	5	45.45%	2	4	Yang (n = 1.5)	[102.302, 111.657]	[0.844, 0.852]	[844.450, 852.380]
	0	2	18.18%	2	5	Yang (n = 1.5)	[111.657, 123.606]	[0.834, 0.844]	[834.420, 844.450]
Semi-humid	0	15	100.00%						
	0	2	13.33%	1	2	Schreiber	[32.268, 38.265]	[0.943, 0.948]	[943.020, 948.690]
	0	6	40.00%	1	3	Schreiber	[38.265, 57.529]	[0.925, 0.943]	[925.021, 943.020]
	0	5	33.33%	1	4	Schreiber	[57.529, 68.321]	[0.915, 0.925]	[915.091, 925.021]
	0	2	13.33%	1	5	Schreiber	[68.321, 76.457]	[0.907, 0.915]	[907.670, 915.091]

* Water balance model (W.B.M). ** A_Index= IAE_a_/IAR. *** Q = (A_Index) × 10^3^.

**Table 6:** Set of rules used in CART algorithm to estimate predicted ARE in five climatic areas, applied in the north of Algeria.

Climate Floor	*p*-Value	Objects	%	Parent Node	Sons Node	W.B.M *	IARR (W.B.M)	A-Index **	Q ***
Mediterranean	0.0371	16	100.00%						
	0	11	68.75%	1	2	Zhang (W = 0.7)	[31.604, 42.121]	[0.913, 0.923 ]	[913.504, 923.161]
	0	5	31.25%	1	3	Zhang (W = 0.7)	[42.121, 53.600]	[0.903, 0.913]	[903.070, 913.504]
	0	6	37.50%	2	4	Sharif	[26.037, 47.666]	[0.878, 0.897]	[878.610, 897.822]
	0	4	25.00%	2	5	Sharif	[47.666, 60.976]	[0.867, 0.878]	[867.011, 878.610]
	0	1	6.25%	2	6	Sharif	[60.976, 61.917]	[0.866, 0.867]	[866.170, 867.011]
Semi-dry	0	45	100.00%						
	0	2	4.44%	1	2	Sharif	[17.101, 21.332]	[0.902, 0.905]	[902.051, 905.882]
	0	3	6.67%	1	3	Sharif	[21.332, 25.534]	[0.898, 0.902]	[898.270, 902.051]
	0	3	6.67%	1	4	Sharif	[25.534, 28.247]	[0.895, 0.898]	[895.831, 898.270]
	0	5	11.11%	1	5	Sharif	[28.247, 31.418]	[0.893, 0.895]	[893.000, 895.831]
	0	10	22.22%	1	6	Sharif	[31.418, 35.862]	[0.889, 0.893]	[889.041, 893.000]
	0	9	20.00%	1	7	Sharif	[35.862, 39.475]	[0.885, 0.889]	[885.833, 889.041]
	0	11	24.44%	1	8	Sharif	[39.475, 48.371]	[0.877, 0.885]	[877.990, 885.833]
	0	2	4.44%	1	9	Sharif	[48.371, 52.023]	[0.874, 0.877]	[874.791, 877.990]

* Water balance model (W.B.M). ** A_Index= IAE_a_/IAR. *** Q = (A_Index) × 10^3^.

**Table 7:** Set of rules used in CART algorithm to estimate predicted IARR in five climatic areas, applied in the north of Algeria.

Climate Floor	Node Son	Condition	IARR-CART
Very humid	Node2	If Q ^1^ (Zhang) ∈ [772.652, 827.330] **or** IARR ^2^ (Zhang) ∈ [141.207, 209.585]	185.00
	Node3	If Q (Zhang) ∈ [723.360, 772.652] **or** IARR (Zhang) ∈ [209.585, 275.501]	307.80
	Node4	If Q (Zhang) ∈ [627.731, 723.360] **or** IARR (Zhang) ∈ [275.501, 417.300]	367.47
	Node5	If (Q (Sharif) ∈ [684.763, 717.950] and Q (Zhang) ∈ [627.731, 723.360]) **or** (IARF (Sharif) ∈ [249.605, 296.940] and IARR (Zhang) ∈ [275.501, 417.300])	241.93
	Node6	If(Q (Sharif) ∈ [648.441, 684.763] and Q (Zhang) ∈ [627.731, 723.360]) **or** (IARR (Sharif) ∈ [296.940, 351.434] and IARR (Zhang) ∈ [275.501, 417.300])	493.00
Humid	Node2	IfQ (Schreiber) ∈ [835.011, 860.960] **or** IARR (Schreiber) ∈ [67.956, 98.557]	113.47
	Node3	If Q (Schreiber) ∈ [828.610, 835.011] **or** IARR (Schreiber) ∈ [98.557, 106.253]	139.25
	Node4	If (Q (Yang) ∈ [844.450, 852.380] and Q (Schreiber) ∈ [835.011, 860.960]) **or** (IARR (Yang) ∈ [102.302, 111.657] and IARR (Schreiber) ∈ [67.956, 98.557])	104.40
	Node5	If (Q (Yang) ∈ [834.420, 844.450] and Q (Schreiber) ∈ [835.011, 860.960]) **or** (IARR (Yang) ∈ [111.657, 123.606] and IARR(Schreiber) ∈ [67.956, 98.557])	136.15
Semi-humid	Node2	IfQ (Schreiber) ∈ [943.020, 948.690] **or** IARR (Schreiber) ∈ [32.268, 38.265]	58.00
	Node3	If Q (Schreiber) ∈ [925.021, 943.020] **or** IARR (Schreiber) ∈ [38.265, 57.529]	78.50
	Node4	If Q (Schreiber) ∈ [915.091, 925.021] **or** IARR (Schreiber) ∈ [57.529, 68.321]	96.80
	Node5	If Q (Schreiber) ∈ [907.670, 915.091] **or** IARR (Schreiber) ∈ [68.321, 76.457]	106.00
Mediterranean	Node2	If Q (Zhang) ∈ [913.504, 923.161] **or** IARR (Zhang) ∈ [31.604, 42.121]	37.75
	Node3	If Q (Zhang) ∈ [903.070, 913.504] **or** IARR (Zhang) ∈ [42.121, 53.600]	49.16
	Node4	If (Q (Sharif) ∈ [878.610, 897.822] and Q(Zhang) ∈ [913.504, 923.161]) **or** (IARR (Sharif) ∈ [26.037, 47.666] and IARR (Zhang) ∈ [31.604, 42.121])	31.42
	Node5	If (Q (Sharif) ∈ [867.011, 878.610] and Q (Zhang) ∈ [913.504, 923.161]) **or** (IARR (Sharif) ∈ [47.666, 60.976] and IARR (Zhang) ∈ [31.604, 42.121])	40.95
	Node6	If (Q (Sharif) ∈ [866.170, 867.011] and Q (Zhang) ∈ [913.504, 923.161]) **or** (IARR (Sharif) ∈ [60.976, 61.917] and IARR (Zhang) ∈ [31.604, 42.121])	62.95
Semi-dry	Node2	IfQ (Sharif) ∈ [902.051, 905.882] **or** IARR (Sharif) ∈ [17.101, 21.332]	7.75
	Node3	If Q (Sharif) ∈ [898.270, 902.051] **or** IARR (Sharif) ∈ [21.332, 25.534]	9.33
	Node4	If Q (Sharif) ∈ [895.831, 898.270] **or** IARR (Sharif) ∈ [25.534, 28.247]	12.94
	Node5	If Q (Sharif) ∈ [893.000, 895.831] **or** IARR (Sharif) ∈ [28.247, 31.418]	14.82
	Node6	If Q (Sharif) ∈ [889.041, 893.000] **or** IARR (Sharif) ∈ [31.418, 35.862]	19.28
	Node7	If Q (Sharif) ∈ [885.833, 889.041] **or** IARR (Sharif) ∈ [35.862, 39.475]	20.87
	Node8	If Q (Sharif) ∈ [877.990, 885.833] **or** IARR (Sharif) ∈ [39.475, 48.371]	28.22
	Node9	If Q (Sharif) ∈ [874.791, 877.990] **or** IARR (Sharif) ∈ [48.371, 52.023]	36.84

^1^ Q= (A_Index) × 10^3^. ^2^ IARR estimated by water balance models.

**Table 8:** Statistical tests of data distribution and performance analysis of proposed models MR, CART, and MR-CART, applied in five climate areas of northern Algeria.

Climate Floor	Parameters	Real Data	MR Model	CART Model	(MR-CART) Model
Very humid	Min	166.000	157.291	185.000	167.580
	Max	497.000	505.874	493.000	509.539
	Mean	274.591	274.512	274.591	274.550
	SD	105.244	99.371	100.403	102.307
	R^2^	1.000	0.899	0.922	0.957
	R^2^_Adj_	1.000	0.893	0.910	0.945
	RMSE	0.000	40.254	33.891	27.537
	MAE	0.000	28.083	23.644	19.211
Humid	Min	95.000	100.494	104.400	98.252
	Max	149.000	145.499	139.250	148.168
	Mean	122.845	123.107	122.845	118.843
	SD	18.355	16.629	16.872	17.628
	R^2^	1.000	0.825	0.851	0.886
	R^2^_Adj_	1.000	0.817	0.845	0.875
	RMSE	0.000	9.204	7.990	7.614
	MAE	0.000	7.684	6.671	6.357
Semi-humid	Min	55.000	57.891	58.000	57.662
	Max	110.000	110.844	106.000	110.433
	Mean	85.533	85.620	85.533	85.506
	SD	15.770	15.290	14.800	15.469
	R^2^	1.000	0.943	0.889	0.958
	R^2^_Adj_	1.000	0.939	0.881	0.949
	RMSE	0.000	4.199	5.849	4.049
	MAE	0.000	3.653	5.089	3.522
Mediterranean	Min	28.000	29.279	31.421	29.463
	Max	62.950	57.507	62.950	58.365
	Mean	41.316	41.229	41.316	41.310
	SD	10.083	8.793	9.231	9.521
	R^2^	1.000	0.772	0.841	0.904
	R^2^_Adj_	1.000	0.764	0.838	0.892
	RMSE	0.000	5.661	4.336	3.678
	MAE	0.000	4.322	3.310	2.808
Semi-dry	Min	7.000	5.524	7.750	6.952
	Max	51.000	35.852	36.845	37.724
	Mean	20.470	20.401	20.470	21.026
	SD	8.349	6.984	7.153	7.271
	R^2^	1.000	0.711	0.720	0.723
	R^2^_Adj_	1.000	0.704	0.714	0.719
	RMSE	0.000	4.648	4.570	4.521
	MAE	0.000	2.148	2.112	2.090

Standard deviation (SD), coefficient of determination (R^2^), adjusted coefficient of determination (R^2^_Adj_), mean absolute error (MAE), root mean square error (RMSE).

**Table 9:** Two sample parametric and non-parametric statistical tests used to compare variability and data distribution of real and predicted IARR that were obtained through a set of water balance models in the northern Algeria area.

Statistic	Real Data	Schreiber	Ol’dekop	Pike	Budyko	Yang	Sharif	Zhang	MR	CART	MR-CART
Min	7.000	0.555	2.039	3.029	1.298	9.552	17.101	6.913	5.524	7.750	6.952
Max	497.00	377.825	237.453	296.477	310.726	384.671	351.434	417.300	505.874	493.000	502.539
Mean	20.625	5.981	6.771	9.950	6.331	25.252	35.629	20.618	21.852	20.867	20.844
1st Q	39.000	18.680	13.995	20.294	16.404	44.170	50.550	38.715	37.485	38.896	38.924
Median	104.25	72.775	41.415	58.088	57.358	104.230	116.995	113.758	105.825	104.400	103.650
3rd Q	81.719	52.926	32.396	44.254	42.916	76.388	84.857	78.989	81.704	81.719	81.716
SD	96.512	74.249	42.718	55.139	58.991	75.245	69.890	85.124	95.493	95.639	95.986
*T*-test	1.000	<0.0001	<0.0001	<0.0001	<0.0001	0.078	0.347	0.290	0.835	0.833	0.845
Z-test	1.000	<0.0001	<0.0001	<0.0001	<0.0001	0.075	0.345	0.287	0.830	0.828	0.844
F-test	1.000	0.009	<0.0001	<0.0001	<0.0001	0.063	0.081	0.209	0.915	0.927	0.939
Sign-test	1.000	<0.0001	<0.0001	<0.0001	<0.0001	0.001	<0.0001	0.421	0.773	0.326	0.773
WSR-test	1.000	<0.0001	<0.0001	<0.0001	<0.0001	0.032	<0.0001	0.447	0.705	0.335	0.721

1st quartile (1st Q), 3rd quartile (3rd Q), standard deviation (SD), Student’s t-test (*T*-test), Fisher’s test, (F-test), Wilcoxon signed-rank test (WSR-test).

## Data Availability

The data presented in this study are available on request from the corresponding author.
